# A Transferrin-Conjugated Hollow Nanoplatform for Redox-Controlled and Targeted Chemotherapy of Tumor with Reduced Inflammatory Reactions: Erratum

**DOI:** 10.7150/thno.72625

**Published:** 2022-04-26

**Authors:** Jun Zhou, Menghuan Li, Wei Qi Lim, Zhong Luo, Soo Zeng Fiona Phua, Runlan Huo, Liqi Li, Ke Li, Liangliang Dai, Junjie Liu, Kaiyong Cai, Yanli Zhao

**Affiliations:** 1 School of Life Science, Chongqing University, Chongqing 400044, P. R. China;; 2 Division of Chemistry and Biological Chemistry, School of Physical and Mathematical Sciences, Nanyang Technological University, 21 Nanyang Link, Singapore 637371;; 3 Key Laboratory of Biorheological Science and Technology, Ministry of Education, Chongqing University, Chongqing 400044, P. R. China;; 4 Department of General Surgery, Xinqiao Hospital, Third Military Medical University, Chongqing 400037, China;; 5 School of Materials Science and Engineering, Nanyang Technological University, 50 Nanyang Avenue, Singapore 639798.

The authors regret that the original version of our manuscript contained some inadvertent errors regarding the representative images in Figure 3e/f and Figure 9. The fluorescent imaging results regarding the uptake of HMSNs-S-S-Tf@FITC within MDA-MB-231 cells after incubation of 12 h (Figure 3f) and HMSNs@FITC at 24 h (Figure 3e1) were erroneously duplicated. Meanwhile, inappropriate images were used for morphological analysis of major mouse organs after H&E staining in Figure 9 due to our careless mistakes during figure assembly. The correct versions of Figure 3 and Figure 9 are shown here below. The authors confirm that the corrections made in this erratum do not affect the original conclusions. The authors apologize for any inconvenience or misunderstanding that the errors may have caused.

## Figures and Tables

**Figure 3:**
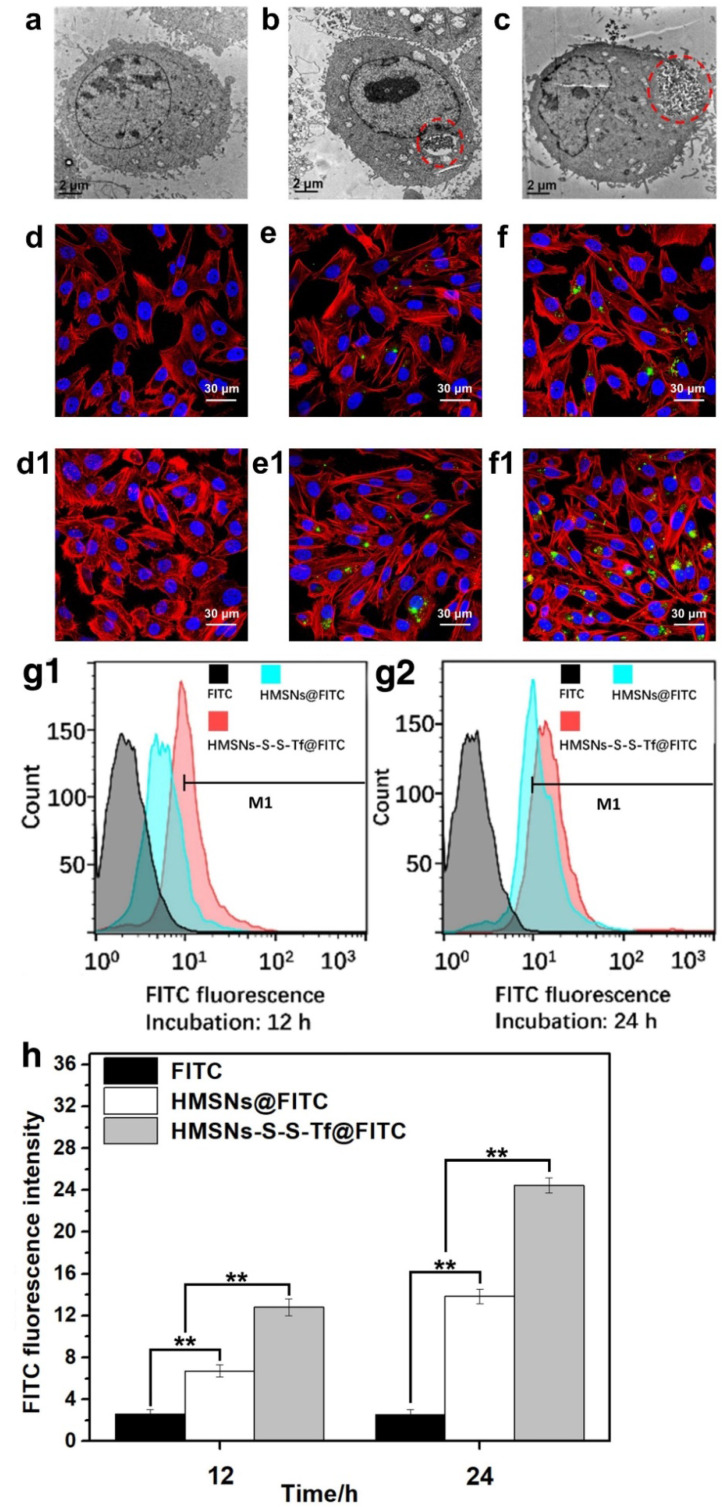
Representative TEM images showing an MDA-MB-231 cell grown on (a, control group) TCPS and those treated with (b) free HMSNs or (c) HMSNs-S-S-Tf after 12 h. Endocytosed nanoparticles were marked with red circles. Scale bar: 2 μm. Representative CLSM images showing the distributions of (d and d1) free FITC, (e and e1) HMSNs@FITC, and (f and f1) HMSNs-S-S-Tf@FITC within MDA-MB-231 cells after incubations of 12 h and 24 h, respectively. Scale bar: 50 μm. Red: cytoskeleton, blue: cell nuclei, green: FITC-loaded nanoparticles. Flow cytometric evaluation of the nanoparticle intake with or without Tf conjugation in panel (g1, 12h) and (g2, 24h), respectively. The results were compared quantitatively in panel (h). (n = 6, **p < 0.01)

**Figure 9:**
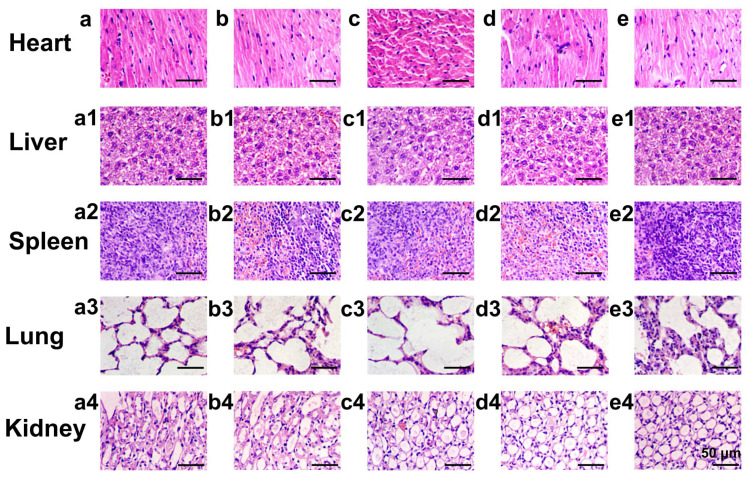
Representative morphologies of vital organs after treatment with (a-a4) saline as a control group, (b-b4) HMSNs, (c-c4) DOX, (d-d4) HMSNs@DOX, or (e-e4) HMSNs-S-S-Tf@DOX for 21 days, respectively. Panel a-e: heart; Panel a1-e1: liver; Panel a2-e2: spleen; Panel a3-e3: lung; Panel a4-e4: kidney.

